# Pregnancy Outcomes in Pregnant Women with HIV on Tenofovir Disoproxil Fumarate (TDF) Compared to Tenofovir Alafenamide (TAF)

**Published:** 2022

**Authors:** Matthew A. Thimm, Alison Livingston, Rosemary Ramroop, Ahizechukwu C. Eke

**Affiliations:** 1 Department of Gynecology & Obstetrics, Johns Hopkins University School of Medicine, 600 N Wolfe St, Baltimore, MD 21287, USA; 2 Division of Maternal Fetal Medicine, Department of Gynecology & Obstetrics, Johns Hopkins University School of Medicine, 600 N Wolfe St, Baltimore, MD 21287, USA

**Keywords:** Tenofovir Disoproxil Fumarate, Tenofovir Alafenamide, Antiretroviral agents, HIV, AIDS, Pregnancy, Weight gain

## Abstract

**Objective:**

Our objective was to assess the safety, efficacy, and pregnancy outcomes of Tenofovir Disoproxil Fumarate (TDF) compared to Tenofovir Alafenamide (TAF) use in pregnant women with HIV (PWLHIV).

**Methods:**

This retrospective cohort study of all women who received prenatal care at a single academic center between January 1^st^ 2015 and June 30^th^, 2020 compared outcomes in PWLHIV using TDF compared to TAF. The primary outcome was weight-gain during pregnancy. Secondary outcomes included CD4 count, viral-load, gestational age at delivery, fetal and neonatal outcomes. Outcomes were analyzed using standard statistical tests. Multivariable linear-regression analysis models accounting for potential confounders were created for primary and secondary outcomes, with beta coefficients (*β*) and associated 95% confidence intervals as the primary measure of effect. Statistical analysis was done with STATA 16.

**Results:**

There were 66 women in the TDF group and 34 women in the TAF group. In the overall cohort, the median (interquartile range, IQR) gestational age at delivery for PWLHIV on TDF and TAF were 38.6 (IQR 37.5–39.4) and 38.1 (31.1–39.1) weeks respectively; and most women (85%) were Black/African American. Compared to PWLHIV on a TDF regimen, women on TAF, on average, gained over 3 kg more weight in the 3^rd^ trimester of pregnancy (*β=*3.20, 95% CI 1.64, 7.97; *p=0.03*). Women in the TAF arm were also more likely to have higher median CD4-count (470 cells/mm^3^ versus 669 cells/mm^3^, *p=0.035*) in the third trimester compared to women on TDF. There were no cases of neonatal/infant HIV or death.

**Conclusion:**

Although TAF use was associated with more weight gain compared to TDF, both regimens appear safe and effective during pregnancy. PWLHIV should be counseled about the potential for weight gain with TAF based regimens during pregnancy.

## Introduction

HIV infection in pregnancy continues to be of significant clinical and public health importance [1,2]. The current statistics published by the World Health Organization (WHO) demonstrate that 38 million people are living with HIV in 2019, and 1.1 million (85%) pregnant women living with HIV received antiretroviral therapy (ARVs) during pregnancy and postpartum [1]. Additionally, 53% of children living with HIV in 2019 are on lifelong ARVs [1]. Expectedly, remarkable progress has been made in preventing mother-to-child transmission of HIV during pregnancy and lactation, and management of pregnant women living with HIV has evolved significantly in the last three decades [3,4].

Since the report of the first therapy for HIV in pregnant women living with HIV in 1994, significant improvements in highly active antiretroviral therapy (HAART) have revolutionized the management of HIV during pregnancy for maternal health and prevention of perinatal transmission [2]. These include the use of combination therapy with several newer ARVs, including integrase inhibitors and nucleoside reverse transcriptase inhibitors (NRTIs) [5,6]. While tenofovir disoproxil fumarate (TDF) and tenofovir alafenamide (TAF), two commonly used NRTI backbone in combination ARV, have similar efficacy, resistance profiles, and mechanisms of action (both prodrugs of tenofovir); they differ in their potency and adverse effect profiles [2,4]. Compared with TDF, TAF rapidly enters peripheral blood mononuclear cells (PBMCs) and other lymphoid tissues following ingestion, resulting in approximately 90% decreased plasma tenofovir (TFV) concentrations [2,7,8]. This pharmacokinetic (PK) characteristic increases the potency and antiviral activity of TAF, and decreases the potential for adverse effects from TAF compared to TDF [2]. From a pharmacodynamic (PD) perspective, TAF’s median TFV concentrations exceeds its 90% effective concentration (EC_90_) 1–2 hours after a single dose, whereas TDF does not exceed this threshold until after approximately 3 days of daily dosing [9]. These pharmacokinetic pharmacodynamic (PKPD) characteristics of TAF may be advantageous in pregnancy in order to cause a rapid decline in HIV viral load to prevent perinatal transmission. Despite these advantages, not many centers use TAF during pregnancy for prevention of perinatal transmission [2]. The clinical impact of these differences between TDF and TAF has not been fully studied during pregnancy.

While TDF and TAF have been studied extensively in non-pregnant populations from a pharmaco-epidemiologic perspective [10–17] there are limited studies of TAF use during pregnancy [18,19] despite the increasing use of TAF fixed dose combination ARVs during pregnancy for treatment of maternal HIV infection and for prevention of perinatal transmission. To date, TDF data in pregnancy has been reassuring, as the Promoting Maternal Infant Survival Everywhere (PROMISE) randomized clinical trial did not identify associations between high TDF exposure, as measured by maternal tenofovir diphosphate (TFV-DP) concentrations in dried blood swabs, and adverse maternal, fetal, and neonatal outcomes [20]. Although TAF use in non-pregnant adults has been associated with weight gain and metabolic syndrome [21,22], only one study has evaluated the maternal and fetal outcomes in pregnant and postpartum women living with HIV on TAF compared to TDF [23]. Data from TAF and TDF pharmacoepidemiologic pregnancy studies are critical because the effectiveness of ARV therapy has to be considered alongside the potential for maternal adverse effects and the risk for congenital anomalies in fetuses of pregnant women living with HIV.

Therefore, the objective of this study was to assess the safety, efficacy, maternal and fetal outcomes of Tenofovir Disoproxil Fumarate (TDF) and Tenofovir Alafenamide (TAF) use in pregnant women living with HIV (PWLHIV).

## Materials and Methods

This was a retrospective cohort study of all cases of pregnant women living with HIV at the Johns Hopkins Hospital HIV in pregnancy (HALO) clinic between January 1^st^, 2015 and June 30^th^, 2020 (5 and half year period). The cohort included pregnant women living with HIV aged 18 to 48 years of age, at ≥ 6 0/7 weeks of gestation and their HIV-exposed neonates. Pregnant women met criteria for inclusion if they used a TDF based regimen [TDF, emtricitabine (*Truvada*); TDF, emtricitabine, efavirenz (*Atripla*); TDF, emtricitabine, rilpivirine (*Complera*); TDF, emtricitabine, elvitegravir, cobicistat (*Stribild*)]; or a TAF based regimen [TAF, emtricitabine (*Descovy*); TAF, emtricitabine, rilpivirine (*Odefsey*); TAF, emtricitabine, elvitegravir, cobicistat (*Genvoya*); TAF, emtricitabine, darunavir, cobicistat (*Symtuza*); TAF, emtricitabine, dolutegravir; and TAF, emtricitabine, bictegravir (*Biktarvy*)] during pregnancy. Women were described as ARV naïve (initiators) if they started taking these ARVs (TDF or TAF) for the first time during pregnancy, or ARV experienced if they had previously been on a TDF or TAF regimen before pregnancy and continued it during pregnancy.

Demographic data, medical history, laboratory testing of mother and infant pairs were collected via chart review and recorded on a standardized form. Data extracted included maternal age, parity, ethnicity, gestational age at enrollment, gestational age at delivery, maternal weight in the first, second and third trimesters of pregnancy, viral load in the first, second and third trimesters of pregnancy, CD4 counts in the first, second and third trimesters of pregnancy, history of alcohol, cocaine, tobacco or heroin use prior to pregnancy, methadone use during pregnancy, hypertension during pregnancy, gestational diabetes mellitus, preterm delivery, Hepatitis B and/or Hepatitis C co-infection during pregnancy, and mode of delivery. Fetal and neonatal outcomes included intrauterine fetal death, the presence of fetal anomalies, birth weight, low birth weight (birth weight <2500 grams), and neonatal death. The exposure of interest was TDF or TAF use, and the primary outcome was maternal weight gain during pregnancy. The secondary outcomes included all other maternal and fetal outcomes. Institutional Review Board (IRB) approval was obtained prior to the study.

### Statistical analysis

The first step in our analysis was exploratory data analysis. We used diagrammatic methods (frequency distributions) to check our data for missing variables, check assumptions, identify outliers and influential observations, determine relationships among the explanatory variables, and assess the direction and approximate size of relationships between explanatory and outcome variables. Descriptive analyses were then performed to describe the study sample. We compared sociodemographic and clinical characteristics, and determined their frequency between the exposure groups (TDF versus TAF). Categorical variables were reported as proportions, and continuous measures were reported as medians as described in Tables 1 and 2. Differences between groups were assessed with Fisher’s exact tests and Chi-square test for categorical variables (where applicable), and Mann Whitney U test for continuous variables.

Linear regression models were used to estimate beta coefficients (*β*) with their associated 95% confidence intervals, accounting for potential confounders – Table 3. *β* regression coefficients indicate how much a dependent variable changes per unit variation of the independent variable, taking into account the other independent variables in the model. For categorical variables (e.g., TDF vs TAF use (TDF=0, TAF=1); nulliparity vs multiparity (nulliparity=0, multiparity=1); no diabetes vs diabetes (no diabetes=0, diabetes=1); and no hypertension versus hypertension (no hypertension=0, hypertension=1), the *β* coefficient represents the effect of moving from the “reference category” (coded as 0) to another category (coded as 1). We assessed model fit and parsimony using Akaike’s Information Criteria (AIC). All statistical analyses were conducted in Stata, version 15.1 (StataCorp, College Station, TX; 2019).

## Results

Table 1 show characteristics of the study population, stratified by NRTI backbone (TDF versus TAF). A total of 100 women were studied (66 women on a TDF based fixed dose combination, and 34 women on a TAF based combination). Pregnant women living with HIV on a TDF regimen were older than those in the TAF arm (median age of 32 years (IQR 29–36) in the TDF group and 29 years (IQR 25–32) in the TAF group; p value= *0.045*). There was no significant difference in parity between the two groups; (*p=0.246*). Gestational age at enrollment (median 14.2 weeks (IQR 11.3–19.6) in the TDF group versus 12.4 weeks (IQR 9.6–16.0) in the TAF group; *p=0.264*) and gestational age at delivery (median 38.6 weeks (IQR 37.5–39.4) in the TDF arm versus 38.1 weeks (IQR 37.1–39.1) in the TAF arm; *p=0.203*) were similar. The majority of participants were Black or African American (54/66 (82 %) in the TDF group and 31/34 (91%) in the TAF group; (*p= 0.115*).

Maternal weight in the first and second trimesters of pregnancy were similar between women on TDF compared to those on TAF (median 81.1 kg (IQR 66.7–98.4) in the TDF arm versus 81.1 kg (IQR 68.1–101.2) in the TAF arm in the first trimester; *p=0.931*; and 81.6 kg (IQR 70–99.3) in the TDF arm versus 81.2 kg (IQR 69.7–103.8) in the TAF arm in the second trimester; *p=0.967*). Women in the TAF arm were more likely to weigh more in the third trimester compared to women on TDF (87.1 kg (IQR 77.2–100.8) in the TDF arm versus 92.1 kg (IQR 79.8–108.9) in the TAF arm; *p=0.042*). Compared to women on TDF, women on a TAF based regimen had a greater weight gain during the third trimester of pregnancy (3.75kg (IQR 1.2–5.7) on TDF versus 4.3kg (IQR 2.7–13.6) on TAF; p=*0.036*). Rates of viral suppression between the two groups were not significantly different; 48/66 (73%) of women on TDF versus 25/34 (74%) of women on TAF had viral loads <20 copies/mL (*p=0.964*) in the first trimester; 54/66 (82%) of women on TDF versus 28/34 (82%) of women on TAF had viral loads <20 copies/mL (*p=0.854*) in the second trimester, and 55/66 (84%) of women on TDF versus 29/34 (86%) of women on TAF had viral loads <20 copies/mL (*p=0.791*) in the third trimester of pregnancy. Women on TAF were more likely than those on a TDF regimen to have a higher CD4 count (median 470 cells/mm^3^ (IQR 355–594) versus 669 cells/mm^3^ (514–750); *p=0.035*) in the third trimester of pregnancy.

There were no significant differences between women on TDF and those on TAF with respect to alcohol, cocaine, heroin, methadone, and tobacco use, hypertension in pregnancy, preterm delivery, hepatitis B and C infections, and mode of delivery. Four women in the TDF arm (6%) were delivered by Cesarean for viral loads >1,000 copies/mL, while none was delivered in the TAF arm solely for a high viral load indication, however this was not statistically significant (*p=0.083*). All women in this study reported >90% medication adherence.

Table 2 shows the fetal and neonatal characteristics in this study population stratified by NRTI backbone (TDF versus TAF). There were no statistically significant differences between women taking TDF versus TAF with respect to birth weight (median neonatal weight of 3230 grams (IQR 2480–3520) in the TDF arm versus 3150 grams (IQR 2580–3495) in the TAF arm; *p=0.260*), intrauterine fetal death, fetal anatomic abnormalities, low birth weight, or neonatal death. None of the infants of the mothers on TDF or TAF were HIV infected. 5/66 fetuses (8%) in the TDF arm and 3/34 fetuses (9%) in the TAF arm had a diagnosis of some fetal anatomic anomalies during pregnancy and postpartum, including mild urinary tract dilatation (two fetuses), unilateral postaxial polydactyly (two fetuses), fetal dangling choroid (one fetus), left supernumerary nipple (one fetus), urachal cyst (one fetus), and right talipes equinovarus (one fetus).

Table 3 shows the univariable and multivariable linear regression analysis of the associations between TDF and TAF use and a number of covariates. In the univariable analysis using logistic regression, pregnant women living with HIV on a TAF regimen, on average, gained 4.5 kg more weight during the 3^rd^ trimester of pregnancy compared to those on TDF (*β=*4.50, 95% CI 1.46, 9.14; *p=0.04*)). In the multivariable linear regression model, pregnant women living with HIV on a TAF regimen, on average, gained 3.2 kg more weight during the 3^rd^ trimester of pregnancy compared to those on TDF (*β=*3.20, 95% CI 1.64, 7.97; *p=0.03*), and remained statistically significant as described in Table 3. The remainder of the maternal covariates were not statistically significant.

## Discussion

We found that pregnant women living with HIV and on a TAF regimen, on average, gained 3.2 kg more weight during the 3^rd^ trimester of pregnancy compared to those on TDF. There were no cases of perinatal transmission of HIV or neonatal death in this cohort.

The increased serum progesterone concentrations, as well as other physiologic changes during pregnancy, lead to increased maternal weight, especially in the third trimester of pregnancy [24–26]. The physiologic weight gain during pregnancy is usually within the recommendations set by the Institute of Medicine (IOM) during pregnancy for optimal maternal and fetal outcomes [27], because weight gain in excess of those recommended by the IOM can be associated with adverse maternal and neonatal outcomes [28,29]. In this study, we reported that pregnant women living with HIV on a TAF regimen, on average, gained 3.2 kg more weight during the 3^rd^ trimester of pregnancy compared to those on TDF. The association between TAF-based regimens and weight gain (when compared to TDF), has been demonstrated in several randomized clinical trials, including the ADVANCE [21] and AMBER [22] randomized controlled trial in non-pregnant adults, and the VESTED trial [30] in pregnant women. These clinical trials demonstrate significant differences in weight gain when comparing TAF based regimens to other ARVs. Data from the ADVANCE trial demonstrated that weight gain in non-pregnant women using a TAF based regimen was approximately 3 kg greater than the weight gain in men (6 kg weight gain in men versus 9 kg weight gain in women) [19]. While the explanation for TAF-associated weight gain in non-pregnant adults stem from several risk factors like a ‘return to health’ effect in those with weight loss due to HIV and its complications, low pretreatment CD4 cell count, high viral load, Black race, genetic differences in metabolism of ARVs and their effects on weight gain, female sex [31], and differences in baseline weight [32]; the causes of TAF-associated weight gain during pregnancy are unknown. Similarly, the clinical consequences and mechanisms that induce these differences in weight between TDF and TAF users during pregnancy remain unknown. Although there is evidence from the DISCOVER randomized clinical trial [9] that TDF may have inhibitory effects on weight gain, which might explain some of the weight differences observed when TDF and TAF based regimens are compared [9], pregnant women were excluded from the DISCOVER randomized clinical trial, so it is difficult to extrapolate these findings to pregnant women. Understanding the clinical consequences of TAF-induced weight gain, and how it is modified during pregnancy by covariates like maternal age, gestational age, parity, race and other maternal and fetal factors are critical research questions, especially in women with class III obesity (body mass index of ≥ 40 kg/m^2^).

Results of this study demonstrated that fetal anatomic anomalies were present in 5 fetuses (8%) of women on a TDF based regimen and 3 (9%) of patients on TAF based regimen. These fetal anomalies are minor, with no clinical significance, and unlikely related to TAF or TDF use during pregnancy because there were no specific organ malformation patterns identified. Therefore, a clear causal association between use of TAF or TDF and fetal anomalies cannot be established. TDF exposure during pregnancy has been associated with reduced neonatal whole-body bone mineral density, decreased mean length-for-age Z-scores, and lower head circumference-for-age Z scores at one year of age in children enrolled in the Surveillance Monitoring for ART Toxicities (SMARTT) cohort study [33], but these findings were of uncertain significance. While there is a dose-response relationship between most drugs and the risk of adverse effects and congenital anomalies [34–37], TAF results in lower systemic concentrations of TFV, and produces higher intracellular concentrations of TFV-DP than TDF. It remains uncertain if higher intracellular TFV-DP concentrations would increase the risk of congenital anomalies and adverse pregnancy outcomes. The HIV Antiretroviral Pregnancy Registry (APR), a project established in 1989 to monitor prenatal ARV exposures and detect potential increases in the risk of teratogenicity, keeps record of fetal anomalies resulting from all ARVs used in pregnant women [38]. Although ARV information from the APR continues to increase, there remains a paucity of data on TAF use during pregnancy. In the IMPAACT P1026s TAF 25 mg and 10 mg boosted with cobicistat study arms [18], congenital anomalies considered possibly related to study drugs involved a small number of women receiving TAF based regimens, so it was difficult to conclude that these congenital anomalies were related to TAF or other ARVs in the fixed dose combinations, or if they were incidental findings. In addition, the number of cases related to TAF reported in the APR at this time is insufficient to draw any reasonable conclusions on the association between TAF and any congenital anomalies. As more data become available, additional information on possible adverse effects and risk of teratogenicity will be gathered [39,40].

One of the limitations of this study is the inability to distinguish weight gain attributable to dolutegravir (DTG) versus TAF when drug combinations of DTG/TAF are used in pregnant women. In the ADVANCE and VESTED trial data that randomized participants to TAF/DTG (arm 1), TDF/DTG (arm 2), and TDF/Efavirenz (arm 3), there was increased weight in participants randomized to the TAF/DTG and TDF/DTG arms. However, because women randomized to the TAF/DTG arm had the highest weight gain, it is plausible that both TAF and DTG contributed to the increased weight, but it is unknown to what extent TAF and/or DTG increased maternal weight. Another potential limitation of this study is the relatively small sample size to enable analysis by sub-groups. With an increased sample size, it may have been possible to show statistically significant differences in serum creatinine concentrations between women who used TDF compared to TAF during pregnancy.

Strengths of this study include the relatively high number of pregnant women living with HIV managed at this single center—greater than what is seen in many institutions in the United States. Furthermore, while TAF is gradually increasingly being used in the management of pregnant women living with HIV in most institutions within and outside the US, its routine use during pregnancy for care of pregnant women living with HIV at this institution enabled us to complete this study. Additionally, given that TAF use in this study was part of routine obstetric care (and outside of a research protocol), the results may be more generalizable to more obstetric practices.

In summary, we found that although TAF use was associated with more weight gain compared to TDF, both regimens appear safe and effective during pregnancy. Pregnant women living with HIV should be counseled about the potential for weight gain with TAF based regimens during pregnancy, especially in the third trimester, as well as behavior modifications to mitigate weight gain with TAF based regimens.

## Figures and Tables

**Table 1:** Maternal demographics for pregnant women on TDF versus TAF.

Variable	Tenofovir Disoproxil Fumarate (TDF) N=66	Tenofovir Alafenamide (TAF) N=34	P-value
Age, median in years (*IQR*)	32 (29–36)	29 (25–32)	0.045
Parity, *n (%)*			0.246
Para 0	9 (14)	9 (27)	
Para 1	23 (35)	11 (32)	
Para 2	20 (30)	11 (32)	
Para 3 or greater	14 (21)	3 (9)	
Gestational age at enrollment, *median (IQR)*	14.2 (11.3–19.6)	12.4 (9.6–16.0)	0.264
Gestational age at delivery, *median (IQR)*	38.6 (37.5–39.4)	38.1 (37.1–39.1)	0.203
Race, n (%)			0.115
Black/African American	54 (82)	31 (91)	
Caucasian	8 (12)	0 (0)	
Hispanic	3 (5)	1 (3)	
Other	1 (1)	2 (6)	
Weight (kg), median (IQR)			
1^st^ trimester	81.1 (66.7–98.4)	81.1 (68.1–101.2)	0.931
2^nd^ trimester	81.6 (70–99.3)	81.2 (69.7–103.8)	0.967
3^rd^ trimester	87.1 (77.2–100.8)	92.1 (79.8–108.9)	0.042
Weight gain (kg), median (IQR)			
2^nd^ trimester	2.75 (0.9–4.5)	3.2 (1.1–5.1)	0.554
3^rd^ trimester	3.75 (1.2–5.7)	4.3 (2.7–13.6)	0.036
Number of mothers with viral load <20 copies/mL, n (%)			
1^st^ trimester	48 (73)	25 (74)	0.964
2^nd^ trimester	54 (82)	28 (82)	0.854
3^rd^ trimester	55 (84)	29 (86)	0.791
CD4+ (cells/mm3), median (IQR)			
1^st^ trimester	569 (373–766)	667 (512–973)	0.050
2^nd^ trimester	444 (353–620)	510 (420–722)	0.230
3^rd^ trimester	470 (355–594)	669 (514–750)	0.035
Serum creatinine (mg/dL), median (IQR)			
1^st^ trimester	0.6 (0.5–0.7)	0.6 (0.4–0.6)	0.725
2^nd^ trimester	0.59 (0.51–0.62)	0.57 (0.5–0.6)	0.598
3^rd^ trimester	0.56 (0.5–0.7)	0.50 (0.4–0.7)	0.048
Alcohol use prior to pregnancy, *n (%)*	1 (2)	2 (5)	0.980
Cocaine use prior to pregnancy, *n (%)*	5 (8)	0 (0)	0.100
Heroin use prior to pregnancy, *n (%)*	2 (3)	1 (3)	0.980
Methadone use in pregnancy, *n (%)*	3 (5)	2 (6)	0.771
Tobacco use prior to pregnancy, *n (%)*	6 (9)	7 (20)	0.105
Hypertension during pregnancy, *n (%)*	5 (8)	3 (9)	0.127
Gestational diabetes mellitus, *n (%)*	2 (3)	2 (6)	0.201
Preterm delivery, *n (%)*	9 (14)	3 (9)	0.496
Hepatitis B, *n (%)*	1 (2)	1 (3)	0.629
Hepatitis C, *n (%)*	3 (5)	0 (0)	0.207
Mode of delivery (n (%)			
Vaginal delivery	41 (62)	17 (50)	0.212
Cesarean delivery	25 (38)	17 (50)	
Cesarean delivery for viral load, *n (%)*	4 (6)	0 (0)	0.083

**Table 2:** Fetal and Neonatal outcomes for pregnant women on TDF versus TAF.

Variable	Tenofovir Disoproxil Fumarate (TDF) N=66	Tenofovir Alafenamide (TAF) N=34	P-value
Intrauterine fetal death, *n (%)*	1 (2)	1 (3)	0.638
Fetal anatomic anomalies, *n (%)*	5 (8)	3 (9)	0.932
Birth weight (grams), *median (IQR)*	3230 (2480–3520)	3150 (2580–3495)	0.260
Low birth weight (Birth weight <2500 grams)	4 (6)	3 (9)	0.145
Infant infection status			
Uninfected by best available data, *n (%)*	66 (100)	34 (100)	-
Neonatal death, *n (%)*	0 (0)	0 (0)	-

IQR – Interquartile range

**Table 3:** Univariable and multivariable regression model.

	Univariable linear regression			Multivariable linear regression		
Outcome: Maternal weight gain in the 3^rd^ trimester	Beta Regression coefficient	95% CI	P-value	Beta Regression coefficient	95% CI	P-value
TAF use	4.50	1.46, 9.14	**0.04**	3.20	1.64, 7.97	**0.03**
Maternal age	−0.28	−0.79, 0.23	0.27	−0.11	−0.66, 0.43	0.68
Gestational age	−0.67	−1.38, 0.04	0.06	−0.35	−1.10, 0.41	0.36
Parity	−2.87	−5.89, 0.14	0.27	−1.91	−5.05, 1.23	0.23
Diabetes	−1.87	−21.9, 18.16	0.86	−1.96	−23.0, 19.08	0.85

* *β* regression coefficients indicate how much a dependent variable changes per unit variation of the independent variable, taking into account the other independent variables in the model. For categorical variables (e.g. TDF vs TAF use (TDF=0, TAF=1); nulliparity vs multiparity (nulliparity=0, multiparity=1); no diabetes vs diabetes (no diabetes=0, diabetes=1); and no hypertension versus hypertension (no hypertension=0, hypertension=1), the *β* coefficient represents the effect of moving from the “reference category” (coded as 0) to another (coded as 1).

## References

[1] WHO. Human Immunodeficiency Virus (HIV). Accessed November 27th 2020, https://www.who.int/news-room/fact-sheets/detail/hivaids

[2] Eke AC, Brooks KM, Gebreyohannes RD, Sheffield JS, Dooley KE, Mirochnick M. Tenofovir alafenamide use in pregnant and lactating women living with HIV. Expert Opinion on Drug Metabolism & Toxicology. 2020 Apr;16(4):333–342. 32125906 10.1080/17425255.2020.1738384 PMC9214649

[3] Chi BH, Mbori‐Ngacha D, Essajee S, Mofenson LM, Tsiouris F, Mahy M, Accelerating progress towards the elimination of mother-to-child transmission of HIV: a narrative review. Journal of the International AIDS Society. 2020 Aug;23(8):e25571. 32820609 10.1002/jia2.25571 PMC7440973

[4] Wassner C, Bradley N, Lee Y. A Review and Clinical Understanding of Tenofovir: Tenofovir Disoproxil Fumarate versus Tenofovir Alafenamide. Journal of the International Association of Providers of AIDS Care. 2020 Jan-Dec;19:2325958220919231. 32295453 10.1177/2325958220919231 PMC7163232

[5] Panel on Treatment of Pregnant Women with HIV Infection and Prevention of Perinatal Transmission. Recommendations for Use of Antiretroviral Drugs in Transmission in the United States. Accessed Accessed 1/11/19, Available at http://aidsinfo.nih.gov/contentfiles/lvguidelines/PerinatalGL.pdf

[6] Eke AC, Olagunju A, Best BM, Mirochnick M, Momper JD, Abrams E, Innovative Approaches for Pharmacology Studies in Pregnant and Lactating Women: A Viewpoint and Lessons from HIV. Clinical Pharmacokinetics. 2020 Oct;59(10):1185–1194. 32757103 10.1007/s40262-020-00915-w PMC7550310

[7] Ray AS, Fordyce MW, Hitchcock MJ. Tenofovir alafenamide: A novel prodrug of tenofovir for the treatment of Human Immunodeficiency Virus. Antiviral Research. 2016 Jan;125:63–70. 26640223 10.1016/j.antiviral.2015.11.009

[8] Eke AC, Shoji K, Best BM, Momper JD, Stek AM, Cressey TR, Population Pharmacokinetics of Tenofovir in Pregnant and Postpartum Women Using Tenofovir Disoproxil Fumarate. Antimicrobial Agents and Chemotherapy. 2021 Feb 17;65(3):e02168–20. 33318014 10.1128/AAC.02168-20 PMC8092509

[9] Mayer KH, Molina JM, Thompson MA, Anderson PL, Mounzer KC, De Wet JJ, Emtricitabine and tenofovir alafenamide vs emtricitabine and tenofovir disoproxil fumarate for HIV pre-exposure prophylaxis (DISCOVER): primary results from a randomised, double-blind, multicentre, active-controlled, phase 3, non-inferiority trial. Lancet (London, England). Jul 25 2020;396(10246):239–254. 10.1016/S0140-6736(20)31065-5 PMC9665936 32711800

[10] McCann K, Moorhouse M, Sokhela S. The ADVANCE clinical trial: Changes from baseline to week 96 in DXA-assessed body composition in TAF/FTC +DTG compared to TDF/FTC+DTG, and TDF/FTC/EFV. presented at: 17th European AIDS Conference; 2019; Basel, Switzerland. Accessed January 20th 2020. http://resourcelibrary.eacs.cyim.com/mediatheque/media.aspx?mediaId=78033&channel=28172

[11] Ruane PJ, DeJesus E, Berger D, Markowitz M, Bredeek UF, Callebaut C, Antiviral activity, safety, and pharmacokinetics/pharmacodynamics of tenofovir alafenamide as 10-day monotherapy in HIV-1-positive adults. Journal of Acquired Immune Deficiency Syndromes (1999). Aug 1 2013;63(4):449–55. 23807155 10.1097/QAI.0b013e3182965d45

[12] Pham HT, Mesplede T. Bictegravir in a fixed-dose tablet with emtricitabine and tenofovir alafenamide for the treatment of HIV infection: pharmacology and clinical implications. Expert Opinion on Pharmacotherapy. Mar 2019;20(4):385–397. 30698467 10.1080/14656566.2018.1560423

[13] Sax PE, DeJesus E, Crofoot G, Ward D, Benson P, Dretler R, Bictegravir versus dolutegravir, each with emtricitabine and tenofovir alafenamide, for initial treatment of HIV-1 infection: a randomised, double-blind, phase 2 trial. The Lancet HIV. Apr 2017;4(4):e154–e160. 28219610 10.1016/S2352-3018(17)30016-4

[14] Gallant J, Lazzarin A, Mills A, Orkin C, Podzamczer D, Tebas P, Bictegravir, emtricitabine, and tenofovir alafenamide versus dolutegravir, abacavir, and lamivudine for initial treatment of HIV-1 infection (GS-US-380–1489): a double-blind, multicentre, phase 3, randomised controlled non-inferiority trial. Lancet (London, England). Nov 4 2017;390(10107):2063–2072. 10.1016/S0140-6736(17)32299-7 28867497

[15] Sax PE, Pozniak A, Montes ML, Koenig E, DeJesus E, Stellbrink HJ, Coformulated bictegravir, emtricitabine, and tenofovir alafenamide versus dolutegravir with emtricitabine and tenofovir alafenamide, for initial treatment of HIV-1 infection (GS-US-380–1490): a randomised, double-blind, multicentre, phase 3, non-inferiority trial. Lancet (London, England). Nov 4 2017;390(10107):2073–2082. 10.1016/S0140-6736(17)32340-1 28867499

[16] Venter WD, Moorhouse M, Sokhela S, Fairlie L, Mashabane N, Masenya M, Dolutegravir plus Two Different Prodrugs of Tenofovir to Treat HIV. The New England Journal of Medicine. Aug 29 2019;381(9):803–815. 31339677 10.1056/NEJMoa1902824

[17] Gallant JE, Daar ES, Raffi F, Brinson C, Ruane P, DeJesus E, Efficacy and safety of tenofovir alafenamide versus tenofovir disoproxil fumarate given as fixed-dose combinations containing emtricitabine as backbones for treatment of HIV-1 infection in virologically suppressed adults: a randomised, double-blind, active-controlled phase 3 trial. The Lancet HIV. Apr 2016;3(4):e158–65. 27036991 10.1016/S2352-3018(16)00024-2

[18] Brooks K, Pinilla M, Shapiro D, Capparelli E, Stek A, Mirochnick M. Pharmacokinetics of tenofovir alafenamide 25 mg with PK boosters during pregnancy and postpartum. Oral abstract presented at: 20th International Workshop on Clinical Pharmacology of HIV, Hepatitis, and Other Antiviral Drugs; 14–16 May 2019 2019; Noordwijk, the Netherlands.

[19] Momper JD, Best B, Wang J, Stek A, Cressey TR, Burchett S, Tenofovir alafenamide pharmacokinetics with and without cobicistat in pregnancy. Journal of The International Aids Society. 2018 Jul 1;21:67–68.

[20] Aizire J, Brooks KM, Mirochnick M, Flynn PM, Butler K, Kiser JJ, Antenatal Intracellular Concentrations of Tenofovir Diphosphate and Emtricitabine Triphosphate and Associations Between Tenofovir Diphosphate and Severe Adverse Pregnancy Outcomes: IMPAACT-PROMISE (1077BF) Trial. Journal of Acquired Immune Deficiency Syndromes (1999). Feb 1 2020;83(2):173–180. 31929405 10.1097/QAI.0000000000002247 PMC7409985

[21] Venter WD, Sokhela S, Simmons B, Moorhouse M, Fairlie L, Mashabane N, Dolutegravir with emtricitabine and tenofovir alafenamide or tenofovir disoproxil fumarate versus efavirenz, emtricitabine, and tenofovir disoproxil fumarate for initial treatment of HIV-1 infection (ADVANCE): week 96 results from a randomised, phase 3, non-inferiority trial. The Lancet HIV. Oct 2020;7(10):e666–e676. 33010240 10.1016/S2352-3018(20)30241-1

[22] Orkin C, Eron JJ, Rockstroh J, Podzamczer D, Esser S, Vandekerckhove L, Week 96 results of a phase 3 trial of darunavir/cobicistat/emtricitabine/tenofovir alafenamide in treatment-naive HIV-1 patients. AIDS (London, England). Apr 1 2020;34(5):707–718. 10.1097/QAD.0000000000002463 31833849

[23] Lockman S, Brummel SS, Ziemba L, Stranix-Chibanda L, McCarthy K, Coletti A, Efficacy and safety of dolutegravir with emtricitabine and tenofovir alafenamide fumarate or tenofovir disoproxil fumarate, and efavirenz, emtricitabine, and tenofovir disoproxil fumarate HIV antiretroviral therapy regimens started in pregnancy (IMPAACT 2010/VESTED): a multicentre, open-label, randomised, controlled, phase 3 trial. Lancet (London, England). Apr 3 2021;397(10281):1276–1292. 10.1016/S0140-6736(21)00314-7 PMC8132194 33812487

[24] Costantine MM. Physiologic and pharmacokinetic changes in pregnancy. Frontiers in Pharmacology. 2014;5:65. 24772083 10.3389/fphar.2014.00065 PMC3982119

[25] Soma-Pillay P, Nelson-Piercy C, Tolppanen H, Mebazaa A. Physiological changes in pregnancy. Cardiovascular Journal of Africa. Mar-Apr 2016;27(2):89–94. 27213856 10.5830/CVJA-2016-021 PMC4928162

[26] Eke AC. An update on the physiologic changes during pregnancy and their impact on drug pharmacokinetics and pharmacogenomics. J Basic Clin Physiol Pharmacol. 2021 Dec 8. 10.1515/jbcpp-2021-0312 PMC9174343 34881531

[27] ACOG Committee Opinion number 315, September 2005. Obesity in pregnancy. Obstetrics and Gynecology. Sep 2005;106(3):671–5. 10.1097/00006250-200509000-00054 16135613

[28] Truong YN, Yee LM, Caughey AB, Cheng YW. Weight gain in pregnancy: does the Institute of Medicine have it right? American Journal of Obstetrics and Gynecology. Mar 2015;212(3):362.e1–8. 10.1016/j.ajog.2015.01.027 25725659

[29] Rogozińska E, Zamora J, Marlin N, Betrán AP, Astrup A, Bogaerts A, Gestational weight gain outside the Institute of Medicine recommendations and adverse pregnancy outcomes: analysis using individual participant data from randomised trials. BMC Pregnancy and Childbirth. Sep 2 2019;19(1):322. 31477075 10.1186/s12884-019-2472-7 PMC6719382

[30] Chinula L, Brummel SS, Ziemba L, Stranix-Chibanda L, Coletti A, Krotje C. Safety and efficacy of TAF vs TDF and DTG vs EFV in pregnancy: IMPAACT 2010 trial. presented at: Conference on Retroviruses and Opportunistic Infections, abstract 130LB; March 8–11, 2020 2020; Boston, Massachusetts. Accessed Accessed April 11th, 2020. https://www.croiconference.org/sessions/safety-and-efficacy-dtg-vs-efvand-tdf-vs-taf-pregnancy-impaact-2010-trial

[31] Bares SH, Smeaton LM, Xu A, Godfrey C, McComsey GA. HIV-Infected Women Gain More Weight than HIV-Infected Men Following the Initiation of Antiretroviral Therapy. Journal of Women’s Health (2002). Sep 2018;27(9):1162–1169. 10.1089/jwh.2017.6717 PMC6148723 29608129

[32] Saag MS, Gandhi RT, Hoy JF, Landovitz RJ, Thompson MA, Sax PE, Antiretroviral Drugs for Treatment and Prevention of HIV Infection in Adults: 2020 Recommendations of the International Antiviral Society-USA Panel. JAMA. Oct 27 2020;324(16):1651–1669. 10.1001/jama.2020.17025 PMC11017368 33052386

[33] Zash RM, Williams PL, Sibiude J, Lyall H, Kakkar F. Surveillance monitoring for safety of in utero antiretroviral therapy exposures: current strategies and challenges. Expert Opinion on Drug Safety. Nov 2016;15(11):1501–1513. 27552003 10.1080/14740338.2016.1226281 PMC5071158

[34] Berard A, Ramos E, Rey E, Blais L, St-Andre M, Oraichi D. First trimester exposure to paroxetine and risk of cardiac malformations in infants: the importance of dosage. Birth Defects Research Part B, Developmental and Reproductive Toxicology. Feb 2007;80(1):18–27. 17187388 10.1002/bdrb.20099

[35] Eke AC, Dooley KE, Sheffield JS. Pharmacologic Research in Pregnant Women - Time to Get It Right. The New England Journal of Medicine. Apr 4 2019;380(14):1293–1295. 30943333 10.1056/NEJMp1815325 PMC9214647

[36] Gilbert-Barness E Teratogenic causes of malformations. Annals of Clinical and Laboratory Science. Spring 2010;40(2):99–114. 20421621

[37] Eke AC, Chalaan T, Shukr G, Eleje GU, Okafor CI. A systematic review and meta-analysis of progestogen use for maintenance tocolysis after preterm labor in women with intact membranes. International Journal of Gynaecology and Obstetrics: The Official Organ of the International Federation of Gynaecology and Obstetrics. 2016 Jan;132(1):11–6. 10.1016/j.ijgo.2015.06.058 PMC9941008 26489489

[38] James JS. HIV & AIDS treatment registry database: public registry now online. AIDS Treatment News. Sep 3 1999;(No 326):3. 11366578

[39] Eke AC, Wang J, Amin K, Shapiro DE, Stek A, Smith E, Fosamprenavir with Ritonavir Pharmacokinetics during Pregnancy. Antimicrobial Agents and Chemotherapy. 2020 Mar 24;64(4):e02260–19. 32015036 10.1128/AAC.02260-19 PMC7179299

[40] Eke AC, McCormack SA, Best BM, Stek AM, Wang J, Kreitchmann R, Pharmacokinetics of Increased Nelfinavir Plasma Concentrations in Women During Pregnancy and Postpartum. The Journal of Clinical Pharmacology. 2019 Mar;59(3):386–393. 30358179 10.1002/jcph.1331 PMC6368469

